# Cefiderocol as a Treatment Option for Stenotrophomonas maltophilia Causing Hospital-Acquired/Ventilator-Associated Pneumonia

**DOI:** 10.7759/cureus.38613

**Published:** 2023-05-05

**Authors:** Muhammad Humayoun Rashid, Syeda Neelam Yamin Bukhari, Aliaa Mousa, Ahmed Ali Aziz, Knkush Hakobyan

**Affiliations:** 1 Internal Medicine, Capital Health Regional Medical Center, Trenton, USA; 2 Internal Medicine, Nishtar Medical University, Multan, PAK

**Keywords:** ventilator-associated pneumonia, hospital-acquired pneumonia, multi-drug resistant bacteria, stenotophomonas maltophilia, cefiderocol

## Abstract

Multidrug-resistant (MDR) gram-negative bacteria have been causing havoc for the healthcare system because of the rarity of the treatment options available. *Stenotrophomonas maltophilia* is a non-fermenting gram-negative bacterium that causes different infections, particularly respiratory tract infections. It displays resistance to many antibiotics (e.g., carbapenems, fluoroquinolones, and trimethoprim-sulfamethoxazole). Cefiderocol is a novel antibiotic which still in the preclinical stages of Food and Drug Administration (FDA) approval for* S. maltophilia*. We present the case of a 76-year-old male with end-stage renal disease (ESRD), intubated for acute hypoxemic respiratory failure due to volume overload and worsening oxygenation, who subsequently developed ventilator-associated pneumonia, found to be due to MDR S*tenotrophomonas maltophilia. *The patient ultimately showed clinical improvement with a 7-day course with a renally adjusted dose of cefiderocol. This shows that cefiderocol can prove to be a potential treatment option against serious infections caused by difficult-to-treat *S. maltophilia.*

## Introduction

Multidrug-resistant (MDR) gram-negative bacteria (GNB), particularly those that are carbapenems resistant, and metallo-β-lactamase (MBL)-producing GNB, have been causing problems in the healthcare system for the past 15 years owing to the lack of therapeutic options against them [1]. Among the new therapeutic options introduced, cefiderocol is a novel siderophore cephalosporin that shows activity against GNB including difficult-to-treat (DTR) phenotypes (i.e., resistant to β-lactams including carbapenems and fluoroquinolones) [2]. *Stenotrophomonas maltophilia (S. maltophilia)* is a non-fermenter, opportunistic, gram-negative bacterium that causes various different infections particularly respiratory tract infections [3]. It displays resistance to many antibiotics (e.g., carbapenems, quinolones and trimethoprim-sulfamethoxazole). Here we encountered a case of ventilator-associated pneumonia caused by a multi-drug resistant strain of *S. maltophilia* that responded to cefiderocol therapy.

## Case presentation

A 76-year-old male with a past medical history of end-stage renal disease (ESRD), hypertension, and pancreatic cancer presented with severe shortness of breath and fever. His vital signs were blood pressure 122/82 mmHg, respiratory rate 35 breaths/min, heart rate 92 beats/min, and oxygen saturation 70% on room air. Chest X-ray showed signs of pulmonary congestion. White blood cell (WBC) count was 7.2 x10^3/μL and platelet count was 397 x10^3/μL. He was found to have acute hypoxemic respiratory failure secondary to volume overload due to acute on chronic renal failure. After some time of continuous positive pressure ventilation, he started to desaturate and had increased work of breathing, therefore, he was intubated and placed on the pressure-regulated volume control (PRVC) mode of the ventilator. Hemodialysis sessions were started on an alternate day basis. Initial sputum and blood cultures were negative.

**Figure 1:**
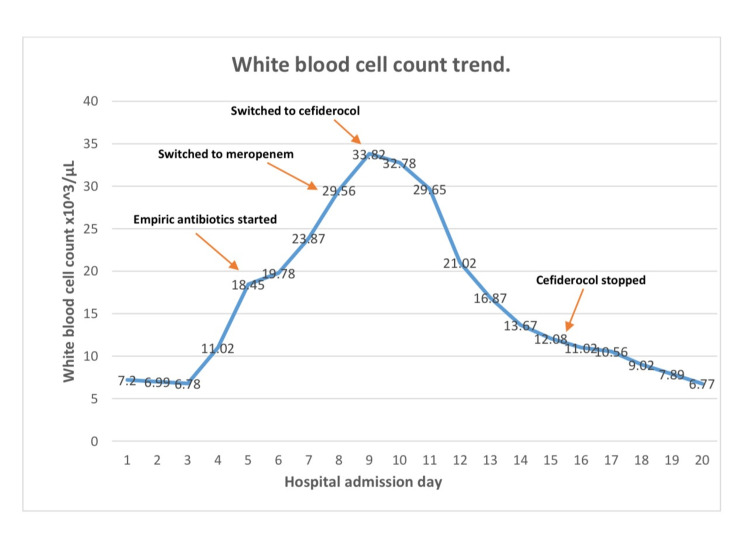
White blood cell count trend throughout hospitalization.

**Figure 2:**
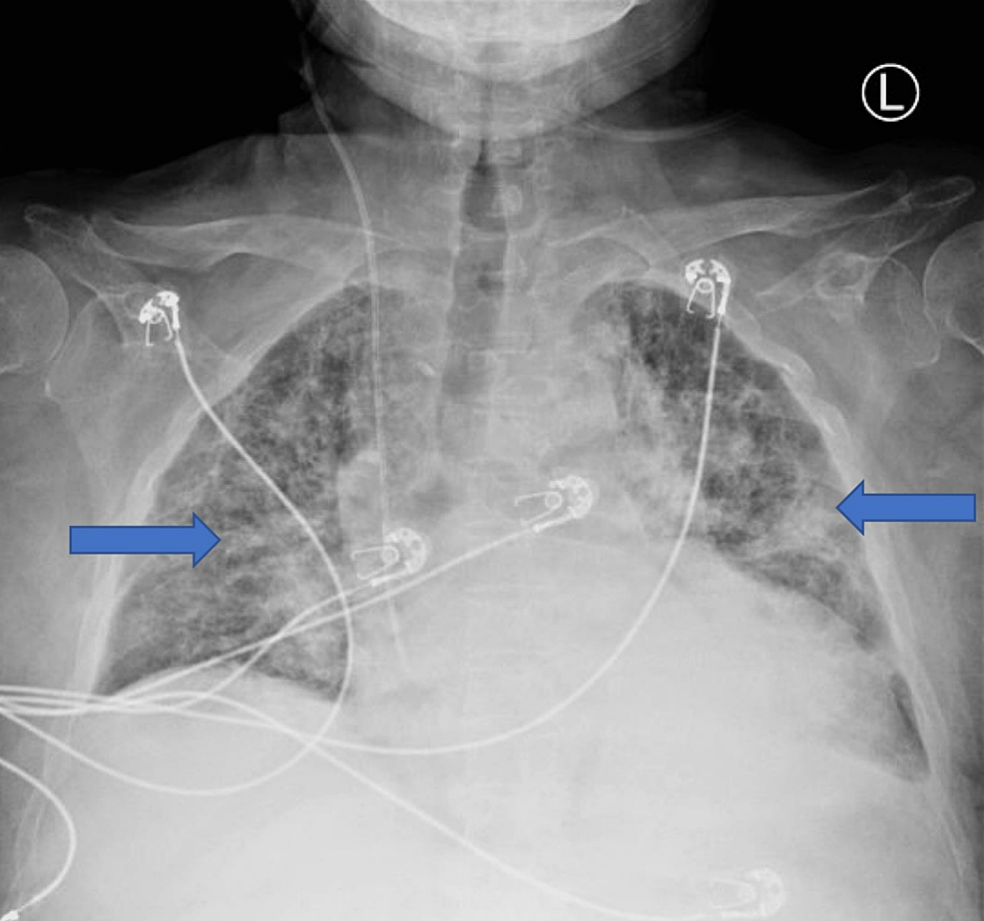
Chest X-ray before cefiderocol treatment. Blue arrows show bilateral interstitial infiltrates.

On day 5 of his hospital stay, he was febrile temperature 38.7 degrees Celsius, WBC count was 18.45 x10^3/μL (Figure 1), predominantly neutrophilia. Chest X-ray showed bilateral interstitial infiltrates as shown by blue arrows in Figure 2.

**Figure 3:**
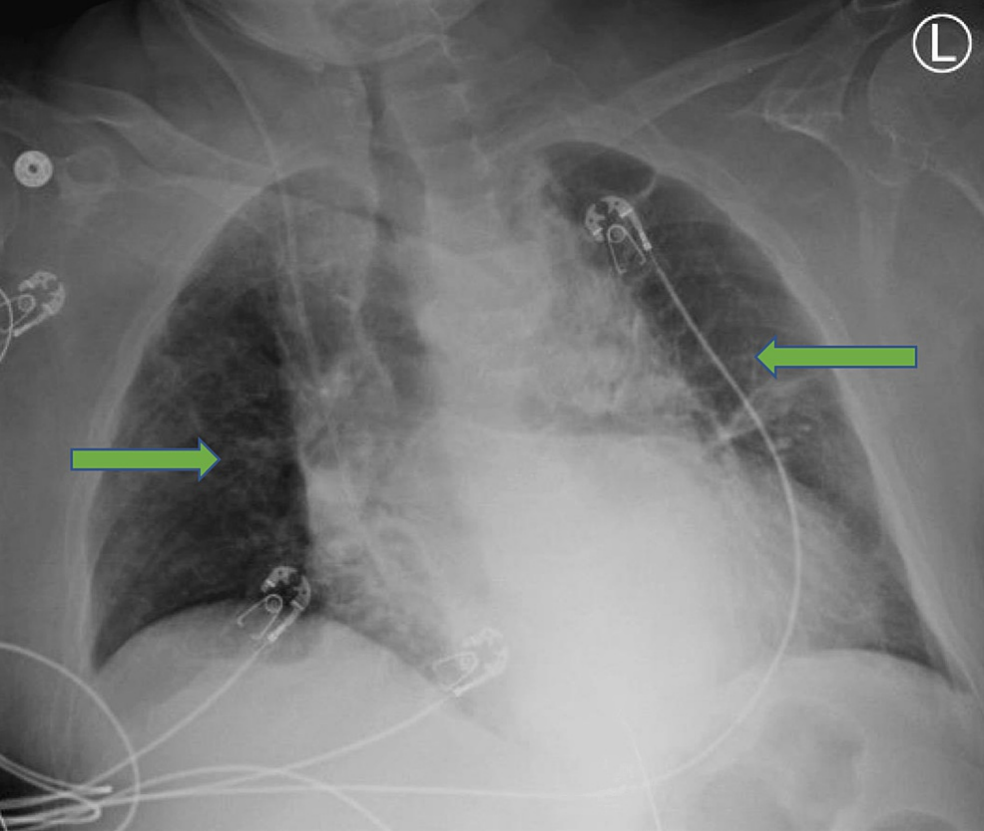
Chest X-ray after 5 days of cefiderocol treatment. Green arrows show reduced interstitial infiltrates after treatment.

Two sets of blood cultures and sputum cultures were sent. The patient was empirically treated with cefepime, and vancomycin based on the likely diagnosis of hospital-acquired pneumonia/ventilator-associated pneumonia (HAP/VAP). Blood cultures did not grow anything over a period of 48 hours, but sputum cultures grew *S. maltophilia* that was resistant to fluoroquinolone, trimethoprim-sulfamethoxazole, cephalosporin, and beta-lactams. Intravenous therapy with meropenem was started on day 7 of hospitalization. The patient's condition deteriorated, WBC count increased, and he developed worsening lung infiltrates. Antibiotic sensitivity test further showed resistance to carbapenems, but sensitivity to cefiderocol. Meropenem therapy was switched to intravenous cefiderocol 750 mg twice daily (the dose was renally adjusted) on day 9 of hospitalization. After 3 days of cefiderocol therapy, his WBC count started to trend down, and his condition improved clinically. Follow-up chest X-rays showed reduced infiltrates as shown in Figure 3.

Repeat sputum cultures on Day 14 of hospitalization were negative. Cefiderocol was stopped on day 16 (a total of 7 days of cefiderocol therapy) and the WBC count kept trending down after that. The trend of WBC count throughout hospitalization in correlation to antibiotic therapy has been shown in Figure 1. He was successfully weaned off from the ventilator and was extubated in a few days.

Due to worsening renal failure and the need for continuous hemodialysis, he remained in the hospital for two more weeks. But unfortunately, he died due to sudden cardiac arrest.

## Discussion

Cefiderocol could play a vital role in fight against the multi-drug-resistant gram-negative bacterial infections. In November 2019 Food and Drug Administration (FDA) approved cefiderocol for the treatment of complicated urinary tract infections. Later, in September 2020, FDA approved it for the treatment of hospital-acquired/ventilator-associated pneumonia caused by *Escherichia coli, Klebsiella pneumoniae, Enterobacter cloacae *complex*, *and *Serratia marcescens*. FDA approval was based on a couple of clinical trials - APEKS-NP [4] and CREDIBLE-CR [5]. *The Lancet Infectious Diseases* have published these trials. The Japanese pharmaceutical company named Shionogi & Co. Ltd. (Osaka) funded these trials and launched the drug named Fetroja.

Richard G. Wunderink, MD led a group of researchers in APEKS-NP, which is a randomized, phase 3, double-blinded, non-inferiority trial that compared meropenem high-dose infusions to cefiderocol for the treatment of gram-negative nosocomial pneumonia [4]. He discovered that at day 14 the all-cause mortality for meropenem was 11.6% (17 out of 146 patients) and for cefiderocol 12.4% (18 out of 145 patients) and for this non-inferiority hypothesis, the adjusted treatment difference was 0.8% with p=0.002 and 95% confidence interval 6.6 to 8.2. He concluded that for the treatment of nosocomial pneumonia caused by gram-negative bacteria, cefiderocol therapy is non-inferior to high-dose meropenem infusion therapy.

In another trial, Matteo Bassetti, MD led a group of researchers in a trial called CREDIBLE-CR [5] which is a randomized, open-label, multicenter, phase 3 trial, in which he studied the efficacy of the cefiderocol therapy compared to the best available therapy (BAT) for the treatment of infections caused by carbapenems-resistant gram-negative bacteria such as bacteremia, sepsis, hospital-acquired pneumonia, and complicated urinary tract infections. He concluded that although the all-cause mortality was slightly high in the cefiderocol group, the clinical efficacy was almost similar in both groups.

For *S. maltophilia,* susceptibility test interpretive criteria are not yet recognized by FDA. In a study including animal models of rat lung and murine thigh, cefiderocol has shown its efficacy in treating *S. maltophilia* infections especially those resistant to empiric antibiotics such as carbapenems and trimethoprim-sulfamethoxazole [6-7]. The results from these animal studies are comparable to the in vitro results. These preclinical trials will prove to be the cornerstone for human trials. We found cefiderocol to be efficacious against *S. maltophilia *in our patient as well. 

Here in our case, we started the patient on broad-spectrum antibiotic therapy empirically after drawing blood and sputum cultures. When the sputum culture identified *S. maltophilia *as a causative agent that was resistant to the empiric treatment, we switched the patient to carbapenem, which is thought to be the treatment of choice for resistant gram-negative pathogens. There was no clinical improvement shown by the patient. Sensitivity tests further showed that this pathogen is also resistant to carbapenems but is sensitive to cefiderocol. Immediately after starting cefiderocol, the patient showed signs of clinical improvement with lower oxygen requirement, better gas exchange, down trending WBC count as shown in Figure 1, and reduced infiltrates in the lungs as shown in Figure 3. This indicated the continuation of therapy. Meanwhile patient had kidney dysfunction and the dose of cefiderocol was renally adjusted, i.e., for CrCl (creatinine clearance) <15 mL/minute: 750 mg every 12 hours. The success of the treatment was confirmed with negative blood cultures post-treatment and clinical improvement.

## Conclusions

*S. maltophilia* is a very dangerous pathogen because of its multi-drug resistant nature and property of causing severe hospital-acquired/ventilator-associated pneumonia. Cefiderocol antibiotic is still in the preclinical stages of approval from the FDA against nosocomial pneumonia caused by resistant *S. maltophilia*. As gram-negative bacteria are becoming more and more resistant, cefiderocol will soon prove to be a potentially useful treatment option against them. 
